# Long Dresses

**Published:** 1875-10

**Authors:** 


### LONG DRESSES.

Our landlady's daughter is a young lady of some pretentions to gentility. She wears her bonnet well back upon her head, which is known by all as a mark of high breeding. She wears her trains very long, as the great ladies do in Europe. To be sure their dresses are so made only to sweep the tapestried floors of chateaux and palaces; and those odious aristocrats of the other side do not go dragging through the mud in silks and satins, but, forsooth, must ride in coaches when in full dress. It is true that, considering various habits of the American people, also the little accidents which the best-kept sidewalks are liable to, a lady who has swept a mile or two of them is not exactly in such a condition that one would care to be her neighbor. But confound the make-believe women we have turned loose in our streets! Where do they come from? Not out of Boston parlors, I trust. Why, there isn't a beast or a bird that would drag its tail through the dirt in the way these creatures do their dresses. Because a queen or a duchess wears long robes on great occasions, a maid of all work or a factory-girl thinks she must make herself a nuisance by trailing long skirts about with her—bah! that's what I call getting vulgarity into your bones and marrow. Making believe what you are not is the essence of vulgarity. Show over dirt is one attribute of vulgar people. If any man can walk behind these women and see what she rakes up as she goes, and not feel squeamish, he has got a strong stomach. I wouldn't let one of them into my room without serving them as David served Saul at the cave in the wilderness—cut off his skirts. Don't tell me that a true lady ever sacrifices the duty of keeping all about her sweet and clean to the wish of making a vulgar show. I won't believe it of a lady. There are some things that no fashion has a right to touch, and cleanliness is one of those things. If a woman wishes to show that her husband or father has money, which she wants and means to spend, but doesn't know how, let her buy a yard or two of silk and pin it to her dress when she goes out to walk, and let her unpin it before she goes into the house.—*Prof. Holmes.*

